# A Novel MIMO–SAR Solution Based on Azimuth Phase Coding Waveforms and Digital Beamforming

**DOI:** 10.3390/s18103374

**Published:** 2018-10-09

**Authors:** Fang Zhou, Jiaqiu Ai, Zhangyu Dong, Jiajia Zhang, Mengdao Xing

**Affiliations:** 1 School of Computer and Information, Hefei University of Technology, Hefei 230009, China; aijiaqiu1985@hfut.edu.cn (J.A.); dzyhfut@hfut.edu.cn (Z.D.); 2 East China Research Institute of Electronic Engineering, Hefei 230088, China; zjjreal@126.com; 3 Institute of Electronic Engineering, Xidian University, Xi’an 710071, China; xmd@xidian.edu.cn

**Keywords:** multiple-input multiple-output synthetic aperture radar (MIMO–SAR), radar system, digital beamforming (DBF), azimuth phase coding (APC), orthogonal waveforms

## Abstract

In multiple-input multiple-output synthetic aperture radar (MIMO–SAR) signal processing, a reliable separation of multiple transmitted waveforms is one of the most important and challenging issues, for the unseparated signal will degrade the performance of most MIMO–SAR applications. As a solution to this problem, a novel APC–MIMO–SAR system is proposed based on the azimuth phase coding (APC) technique to transmit multiple waveforms simultaneously. Although the echo aliasing occurs in the time domain and Doppler domain, the echoes can be separated well without performance degradation by implementing the azimuth digital beamforming (DBF) technique, comparing to the performance of the orthogonal waveforms. The proposed MIMO–SAR solution based on the APC waveforms indicates the feasibility and the spatial diversity of the MIMO–SAR system. It forms a longer baseline in elevation, which gives the potential to expand the application of MIMO–SAR in elevation, such as improving the performance of multibaseline InSAR and three-dimensional SAR imaging. Simulated results on both a point target and distributed targets validate the effectiveness of the echo separation and reconstruction method with the azimuth DBF. The feasibility and advantage of the proposed MIMO–SAR solution based on the APC waveforms are demonstrated by comparing with the imaging result of the up- and down-chirp waveforms.

## 1. Introduction

Synthetic aperture radar (SAR) is a powerful remote sensing technique independent of weather and sunlight illumination. With multiple transmitters and multiple receivers employed, multiple-input multiple-output SAR (MIMO–SAR) enables not only the acquisition of additional phase centers and long baselines for high-resolution wide-swath (HRWS) SAR imaging [1,2,3], but also SAR applications like multibaseline interferometry or three-dimensional imaging [4,5,6,7,8]. Moreover, it enables the possibility to utilize multiple SAR observing modes simultaneously in one MIMO–SAR system.

Design of the transmitted waveforms from multiple transmitters is one of the most important and challenging issues in MIMO–SAR implementation. To this end, time-division multiplexing (TDM) waveforms [9,10,11], frequency-division multiplexing (FDM) waveforms [12,13] and code-division multiplexing (CDM) waveforms [14,15,16,17,18] have been proposed in recent decades. TDM uses a time filter, FDM uses a bandpass filter, while the CDM usually uses a matched filter for the reliable separation of radar echoes. In those waveforms, the CDM waveforms, especially the orthogonal waveforms [14,15,16,17,18], are widely discussed for their capacity to obtain a high-resolution wide-swath SAR image. However, those transmitted orthogonal signals share the same frequency band. Thus, the signal energies of all unmatched waveforms are present simultaneously in the focused signal, which will degrade the performance of most MIMO–SAR systems [19,20,21]. For this reason, these mutually orthogonal waveforms do not suit the senior implementation of the MIMO–SAR system very well.

As a solution to this challenge, the azimuth phase coding (APC) waveforms have been presented in the literature, References [22,23], where the Doppler bandpass filter can enable reliable separation of the echoes corresponding to each transmitted waveform. However, the main drawback of the APC technique in [22,23] is that the pulse repetition frequency (PRF) needs to be K-times as large as the Doppler bandwidth, where K is the number of the simultaneously transmitting waveforms. That drawback will lead to a significant reduction of the swath coverage and will limit the application in spaceborne SAR. Thus, finding a way to reduce the PRF is necessary and beneficial.

By transmitting a set of short-term shift-orthogonal waveforms, the radar echoes can be separated by the digital beamforming (DBF) technique [24], which makes the reduction of the PRF possible. Krieger et al. introduced a set of short-term shift-orthogonal waveforms and the DBF technique in elevation in [25], which put up an effective and inspiring approach to the echo separation for MIMO–SAR.

In this paper, a novel approach to apply the APC waveforms in combination with the DBF technique in the azimuth is proposed as a new MIMO–SAR solution, named APC–MIMO–SAR. The simultaneously transmitted APC waveforms are modulated to carry a set of phases, which makes the Doppler of the received signal distinguishable. By utilizing an antenna with several additional azimuth-displaced phase centers [26], the echoes can be well separated by the DBF technique. Additionally, the spatial diversity of the MIMO–SAR can be restored. In this case, the PRF of this MIMO–SAR system just needs to be slightly larger than the Doppler bandwidth [27,28]. The proposed APC waveforms, together with the azimuth DBF, can be used to exploit the potentials of MIMO–SAR without performance degradation.

This paper is organized as follows. In Section 2, the proposed MIMO–SAR solution with the APC waveforms is introduced in detail. Section 3 proposes an echo separation method by the azimuth DBF processing. Section 4 discusses the signal-to-noise ratio and the requirement of the antenna array size. Section 5 presents the simulations on both the point target and distributed targets to show the advantage of the APC–MIMO–SAR and the effectiveness of the proposed solution. Section 6 draws the conclusions.

## 2. Methods

This section describes the aim, architecture and advantage of the proposed APC–MIMO–SAR solution. The architecture of the MIMO–SAR system is introduced first, and then the modulated APC waveform adopted in this paper is analyzed. Finally, we discuss new problems in the signal model and Doppler spectra.

### 2.1. Architecture for Proposed MIMO–SAR System

A novel instrument architecture for transmitting and receiving in the MIMO–SAR has been discussed in detail in this section. This architecture can provide a longer baseline in elevation, which is of benefit to expand the application of MIMO–SAR.

Assuming that the whole aperture is divided into N × M sub-apertures, N in elevation and M in the azimuth. K sub-apertures in the first column are chosen to be the transmitters, and all the sub-apertures are chosen to be the receivers. Thus, there are K transmitters and N × M receivers, apparently K ≤ N. The N × M receiving sub-apertures can receive the echoes simultaneously. The *k*-th transmitting sub-aperture, in the *k*-th row and the first column, is represented as Txk (k = 1, 2, … ,K), while the *nm*-th receiving sub-aperture, in the *n*-th row and the *m*-th column, is represented as Rxnm(n = 1, 2, …, N, m =1, 2, …, M). For separating the echoes in the following processing, plenty of spatial degrees of freedom are needed, which means the azimuth receivers’ number M should be not smaller than the transmitters’ number N (N ≤ M).

**Figure 1:**
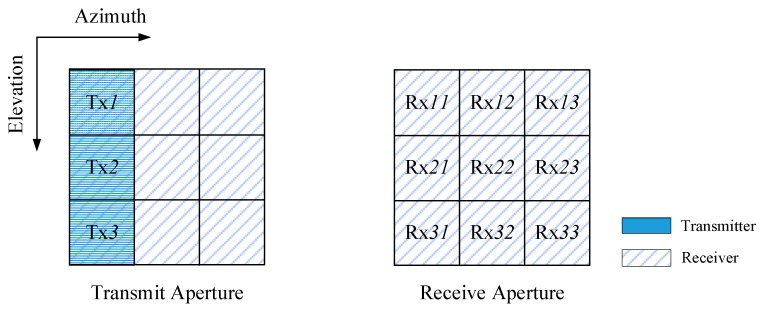
One of the possible architectures of the transmitting and receiving aperture (*K* = 3, *N* = 3, *M* = 3).

Since the architecture of the proposed MIMO–SAR system is diversiform, one possible architecture of the transmitting and receiving aperture is shown in Figure 1. For convenience, Figure 1 gives a 3 × 3 sub-aperture.

**Figure 2:**
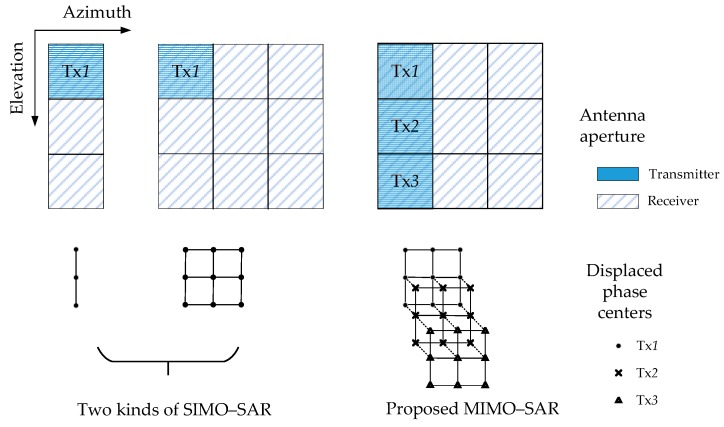
Sketch of the elevation- and azimuth-displaced phase centers.

The proposed MIMO–SAR system architecture can provide additional displaced phase centers [26] both in elevation and azimuth, comparing to a SIMO (single-input multi-output) SAR system. Figure 2 shows the sketch of the elevation- and azimuth-displaced phase centers. In Figure 2, the antenna aperture and the displaced phase centers of two kinds of SIMO–SAR systems are listed on the left, comparing to those of the proposed MIMO–SAR system on the right. Though the displaced phase centers of the proposed MIMO–SAR system are in the same plane, we disassemble them into three layers just for a clearer expression. The first layer marked with “•” corresponds to transmitter Tx1, the second one marked with “×” corresponds to transmitter Tx2, and the third one marked with “△” corresponds to transmitter Tx3. The dotted lines connecting different layers indicate that those phase centers are in the same position on the original plane.

**Figure 3:**
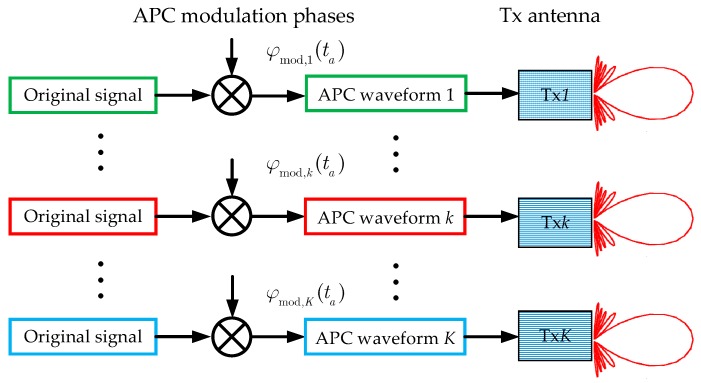
Sketch of the modulated azimuth phase coding (APC) waveforms.

From Figure 3, it is easy to conclude that the long baseline in elevation is formed, doubling the length of the baseline in elevation in the SIMO–SAR system. The longer baseline in elevation provides the additional elevation-displaced phase centers, which can be used to obtain additional degrees of distance freedom. This benefits the MIMO–SAR processing in two aspects. One is to separate echoes and suppress range ambiguities [29,30,31], and the other is to extend the application into multibaseline InSAR [32] and three-dimensional SAR imaging [33]. The additional azimuth-displaced phase centers provide more spatial degrees of freedom, which will make the separation of the echoes’ overlapped spectra via the DBF processing in the azimuth possible [34,35]. This is the new aspect in our paper, which can be used to exploit the potentials of MIMO–SAR without performance degradation.

### 2.2. Modulated APC Waveforms

The APC technique is conceived for single transmit antenna SAR systems to suppress range ambiguities [29,30,31], and it is applied to MIMO–SAR for the design of APC waveforms proposed by Cristallini et al. [22] and Meng et al. [23]. This generation of APC waveforms is proposed to modulate an individual azimuth phase on the original transmit signal for each transmitting aperture, and to ensure the Doppler spectra of echoes from different transmitting apertures occupy different Doppler bands without overlapping each other. Both the conventional chirp signal and the orthogonal waveforms can be used as the original signal/signals to generate a class of the APC waveforms. In the receivers, the Doppler bandpass filter can be used to separate the echoes reliably from different transmit antennas in the Doppler domain. However, the PRF needs to be at least K-times as large as the Doppler bandwidth to get an unaliasing Doppler spectrum, when transmitting K kinds of modulated APC waveforms simultaneously. The overclaim on PRF in [22] and [23] significantly narrows the swath coverage and limits the waveforms’ application, especially in spaceborne MIMO–SAR. To solve this problem, a novel approach to apply the APC waveforms mentioned above is proposed in this section, named APC–MIMO–SAR. The APC–MIMO–SAR receives the echoes by the azimuth sub-apertures to get additional azimuth-displaced phase centers, which makes the reduction of the PRF possible.

Figure 3 shows the scheme of the generation of the modulated APC waveforms. An APC–MIMO–SAR system with K transmitting sub-apertures can transmit K kinds of modulated APC waveforms simultaneously. The APC modulation phase for the *k*-th (k =1, 2, …, K) transmit waveform (Txk) is given by

(1) $$\varphi_{\mathrm{mod},k}(l)=\exp\left(j\frac{\pi }{K}{\left(l+k-1\right)}^{2}\right)\;$$

where l denotes the sequence number of the azimuth pulse. Apparently, l can be expressed as $l=t_{a}f_{PRF}$, where $f_{PRF}$ denotes the pulse repetition frequency (PRF) and $t_{a}$ denotes the azimuth slow time. Thus, substituting $l=t_{a}f_{PRF}$ into (1), the APC modulation phase can be rewritten as

(2) $$\varphi_{\mathrm{mod},k}(t_{a})=\exp\left(j\frac{\pi }{K}{\left(t_{a}f_{PRF}+k-1\right)}^{2}\right)\;$$

**Figure 4:**
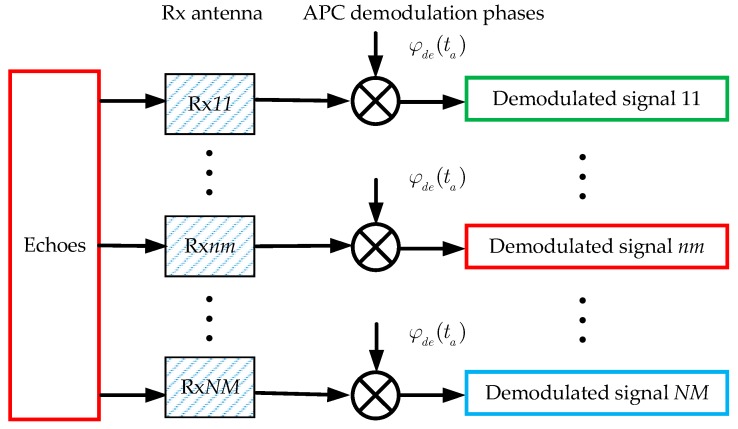
Sketch of the demodulated APC waveforms.

There are N × M receiving sub-apertures which can receive echoes simultaneously, as Figure 4 shows. In order to separate the echoes, the receiver needs to do a demodulating processing after receiving. The APC demodulation phases for each of the receiving sub-apertures are defined as

(3) $$\varphi_{de}(t_{a})\;=\;\exp\left(-j\frac{\pi }{K}t_{a}^{2}f_{PRF}^{2}\right)\;$$

Obviously, the APC demodulation phase is a function which does not vary with the sequence number Rx*nm* of the receiving sub-aperture.

From Figure 3 and Figure 4, one can notice the convenience of the transmitting and receiving of the APC waveforms. The APC modulation and demodulation can be done by adding a multiplier to the transmitter and receiver, instead of redesigning the signal generation module.

After the APC modulation and demodulation, the residual phase of the *k*-th APC waveform can be expressed as

(4) $$\begin{array}{ll} \varphi_{res,k}(t_{a}) & =\varphi_{\mathrm{mod},k}(t_{a})\varphi_{de}(t_{a})\\ & =\exp\left(j2\pi \frac{(k-1)f_{PRF}}{K}t_{a}\right)\exp\left(j\pi \frac{{\left(k-1\right)}^{2}}{K}\right) \end{array}$$

From (4), one can notice that the residual phase is in connection with the APC waveform order k. The first exponential term is a linear phase of the azimuth slow time ta, and the second exponential term is a constant phase. The first exponential term will be expressed as an additional Doppler shift in the azimuth Doppler domain, and the additional Doppler shift frequency $\Delta f_{d,k}$ can be written as

(5) $$\Delta f_{d,k}=\frac{(k-1)f_{\mathrm{PRF}}}{K}\;$$

From (5), it is explicit that there is no additional Doppler shift for the first APC waveform (when *k* = 1). The APC demodulation phase in (3) is constructed based on this strategy. Though the echoes alias in the time domain, they can be separated in the Doppler domain, which will be discussed in Section 3.

### 2.3. Signal Model and Doppler Spectra

The analytic transmitted signal of Txk can be expressed as

(6) $$s_{k}(\tau ,t_{a})=s(\tau ,t_{a})\varphi_{\mathrm{mod},k}(t_{a})\;$$

where τ denotes the fast time, t_a_ denotes the slow time, and $s(\tau ,t_{a})$ is defined as the original signal without the APC modulation phase, which is similar to the echo signal of a monostatic SAR system. For a narrowband transmitted linear frequency modulated (LFM) pulse signal, the modulation form of $s(\tau ,t_{a})$ can be expressed as

(7) $$s(\tau ,t_{a})=w_{r}(\tau )w_{a}(t_{a})\exp(j\pi \gamma \tau^{2})\exp(j2\pi f_{c}\tau )\;$$

where $w_{r}(\cdot )$ is the range window function, $w_{a}(\cdot )$ is the antenna azimuth pattern modulation, e is the chirp rate, and f_c_ is the fundamental carrier frequency.

For a point scatterer in the terrain, the echoes received by Rxnm in the *n*-th row and the *m*-th column can be expressed as

(8) $$s_{nm}(\tau ,t_{a},X_{nm})=\sum_{k=1}^{K}\sigma s(\tau -t_{nm},t_{a}+X_{nm}/2v)\varphi_{res,k}(t_{a}+X_{nm}/2v)\;$$

where σ denotes the backscattering coefficient of the scatterer, *v* denotes the velocity of the radar planform, *X_nm_* denotes the azimuth coordinate of Rxnm. Here, *t_nm_* refers to the propagation time between the radar and the scatterer, which can be calculated by the round-trip slant range from the transmitter *k*Txk to the scatterer, and then back to the receiver Rxnm.

After performing an azimuth Fourier transform, the signal in (8) can be expressed in the Doppler domain as

(9) $$S_{nm}(\tau ,f_{a},X_{nm})=\sum_{k=1}^{K}S(\tau -t_{nm},f_{a}-\Delta f_{d,k})\exp(j2\pi \left(f_{a}-\Delta f_{d,k}\right)X_{nm}/2v)\;$$

where $S(\tau ,f_{a})$ is the Doppler spectrum of $s(\tau ,t_{a})$, $f_{a}$ is the azimuth Doppler frequency, and $\Delta f_{d,k}$ is the additional Doppler shift frequency shown in (5). Thus, the Doppler spectrum of the *k*-th APC waveform can be regarded as its corresponding original signal with a Doppler shift by $\Delta f_{d,k}$.

**Figure 5:**
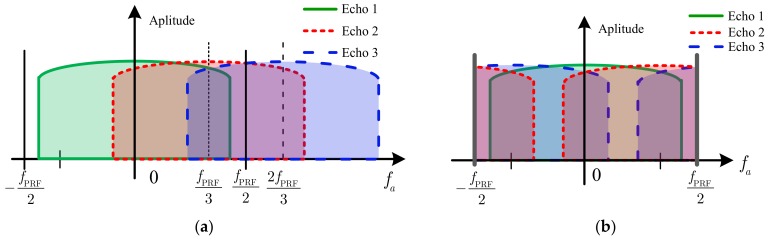
Doppler spectra of echoes of APC waveforms (*K* = 3) before (**a**) and after (**b**) sampling with $f_{PRF}$.

In order to obtain a relatively wide swath, the PRF is set to be slightly higher than the Doppler bandwidth, which will cause the shifted Doppler spectra of the APC waveform echoes to span the neighboring PRF. Thus, after the PRF sampling, the Doppler spectra aliasing will occur, as Figure 5 shows.

When the number of the APC waveforms is 3 (*K* = 3), the Doppler spectrum of the received signal $S_{nm}(\tau ,t_{a},X_{nm})$ is shown in Figure 5. Echo 1, Echo 2 and Echo 3 denote the echoes of the first, second and third APC waveforms, respectively. Figure 5a shows the Doppler spectra before the PRF sampling, where the spectrum spans the neighboring PRF. Figure 5b shows the Doppler spectra after the PRF sampling, where the spectrum aliasing occurs. The aliasing causes the energy accumulation to degrade during the imaging processing, which may make it impossible to get an excellent focused image. Therefore, the Doppler spectrum of each APC waveform echo needs to be separated completely before the imaging processing for the APC–MIMO–SAR.

## 3. Echo Separation and Reconstruction

As the analysis mentioned above, some new problems occur in the proposed APC–MIMO–SAR. The echo signal received simultaneously aliases in the time domain while the one sampled by PRF aliases in the Doppler domain. To deal with these problems, we use the APC technology and the azimuth DBF processing to separate the waveforms, suppress the Doppler ambiguity and reconstruct the echo signal. The approach is discussed in detail below.

Firstly, in a SAR system, it is explicit that the angle–Doppler relation of azimuth Doppler frequency $f_{a}$ and azimuth squint instantaneous angle θ can be described by

(10) $$\sin\theta =\frac{\lambda }{2v}f_{a}\;$$

where λ is wavelength.

**Figure 6:**
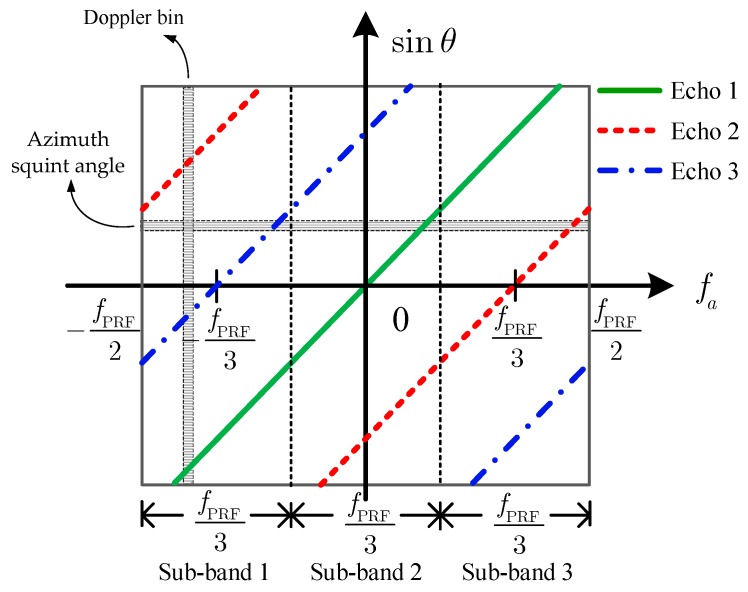
Sketch of the angle–Doppler relation (*K* = 3).

Figure 6 shows the sketch of the angle–Doppler relation of the received signal of the APC waveforms after being demodulated in the azimuth. The sketch provides a concise visualization of the time–frequency relation of the received signal.

From Figure 6, it becomes clear that different APC echo signals present different additional Doppler shift frequencies due to the APC modulation and demodulation. This provides the possibility to separate the time domain-aliased APC waveforms in the azimuth Doppler domain. The angle–Doppler relation of the echo k can be deduced as

(11) $$\sin\theta_{k}(f_{a})=\frac{\lambda }{2v}(f_{a}-\Delta f_{d,k})\;$$

Substituting (11) into (9), the signal in the Doppler domain can be rewritten as

(12) $$S_{nm}(\tau ,f_{a},X_{nm})=\sum_{k=1}^{K}S(\tau -t_{nm},2v\sin\theta_{k}/\lambda )\exp(j2\pi X_{nm}\sin\theta_{k}/\lambda )\;$$

In the case that the PRF is slightly larger than the Doppler bandwidth, it is also shown in Figure 6 that the Doppler spectra of Echo 2 and Echo 3 alias due to the PRF sampling. Thus, the signal of each Doppler bin can be regarded as a sum of echoes of K APC waveforms where different echoes correspond to different azimuth squint angles. Furthermore, each azimuth squint angle corresponds to K APC waveforms, which makes the direct application of the azimuth DBF processing impossible.

To deal with this problem in the APC–MIMO–SAR, a spatial–temporal filtering can be used to extract the signal from each angle. Firstly, the whole Doppler band has been divided uniformly into K sub-bands as the vertical dotted lines shown in Figure 6. The *i*-th (i=1,2,…,K) Doppler sub-band can be described by

(13) $$\left(-\frac{1}{2}+\frac{i-1}{K}\right)f_{\mathrm{PRF}}\leq f_{a}\leq \left(-\frac{1}{2}+\frac{i}{K}\right)f_{\mathrm{PRF}}\;$$

where i denotes the order number of the Doppler sub-band. In each Doppler sub-band, the angle–Doppler relation of each APC waveform has one-to-one correspondence. In this case, the angle–Doppler relation of the echo k in the *i*-th Doppler sub-band can be revised to

(14) $$\sin\theta_{i,k}(f_{a})=\frac{\lambda }{2v}(f_{a}-\Delta f_{d,k}+M_{i,k}f_{\mathrm{PRF}})\;$$

where $M_{i,k}=\left\{ \begin{array}{l} 0,k\leq i\\ 1,k>i \end{array}\right.$ is the Doppler ambiguity number.

**Figure 7:**
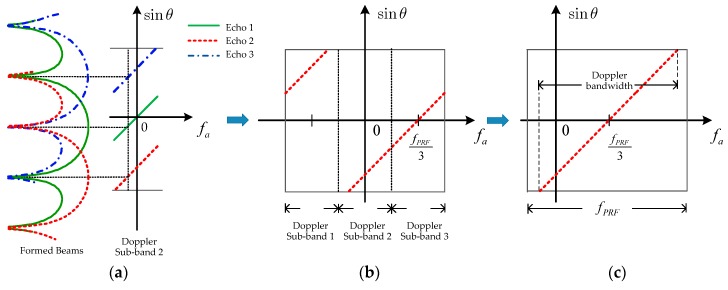
Azimuth digital beamforming for echo (**a**) spatial–temporal filtering, (**b**) separation and (**c**) reconstruction.

Secondly, the spatial–temporal filtering is designed with the azimuth DBF technique [36,37]. The filtering is a weighting operation of MIMO–SAR echo for every Doppler bin of a Doppler sub-band. The wanted echo k in the *i*-th Doppler sub-band without the Doppler ambiguity can be extracted from each angle as Figure 7 shows, and be written as

(15) $$S_{un-amb,i,k}(\tau ,f_{a}-\Delta f_{d,k}+M_{i,k}f_{\mathrm{PRF}})=S(\tau ,f_{a},X_{nm})w(f_{a})\;$$

where $S_{un-amb,i,k}(\tau ,f_{a}-\Delta f_{d,k}+M_{i,k}f_{\mathrm{PRF}})$ denotes the extracted echo, $w(f_{a})$ denotes the weight vector, and $S(\tau ,f_{a},X_{nm})$ denotes the received signal vector. $S(\tau ,f_{a},X_{nm})$ is a 1 × K-dimensional vector constructed by the signal from the *n*-th row receiving sub-apertures, expressed as

(16) $$S(\tau ,f_{a},X_{nm})=\left[S_{n1}(\tau ,f_{a},X_{n1}),\cdots ,S_{nk}(\tau ,f_{a},X_{nk}),\cdots ,S_{nK}(\tau ,f_{a},X_{nK})\right]\;$$

where the exact expression of $S_{nk}(\tau ,f_{a},X_{nk})$ has been shown in (12).

The weight vector can be obtained by solving the following equations

(17) $$w(f_{a})=A^{-1}(f_{a})e_{k}\;$$

where superscript −1 denotes the matrix inverse and $e_{k}={\left[e_{1},\cdots ,e_{q},\cdots ,e_{M}\right]}^{T}$ is a unit vector, and $e_{q=k}=1$, $e_{q\neq k}=0$, which means in vector $e_{k}$ only one element is 1 and not 0. In (17), $A(f_{a})=\left[a_{1}(f_{a}),\ldots ,a_{k}(f_{a}),\ldots ,a_{K}(f_{a})\right]$ is an M × K-dimensional matrix, and $a_{k}(f_{a})$ is the azimuth sub-aperture array steering vector which can be constructed as

(18) $$a_{k}(f_{a})={\left[\exp\left(j2\pi X_{n1}\sin\theta_{k}/\lambda \right),\ldots ,\exp\left(j2\pi X_{nM}\sin\theta_{k}/\lambda \right)\right]}^{T}\;$$

where superscript T denotes the matrix transposition.

As shown in Figure 7a,b, for every Doppler bin in the second Doppler sub-band, the wanted echo can be extracted by steering the formed beam center to the corresponding angle. It is clear that the echo of K APC waveforms can be separated completely via performing the same process on each Doppler sub-band. 

Then, the extracted echo signals from different Doppler sub-bands should be rearranged to reconstruct the whole Doppler band signal, as Figure 7c shows, after compensating the residual phase of the *k*-th APC waveform in (4).

At last, a 2-D focused APC–MIMO–SAR image of the wanted echo k is obtained via the range matching filtering and azimuth focusing processing, which can be applied to the subsequent applications.

**Figure 8:**
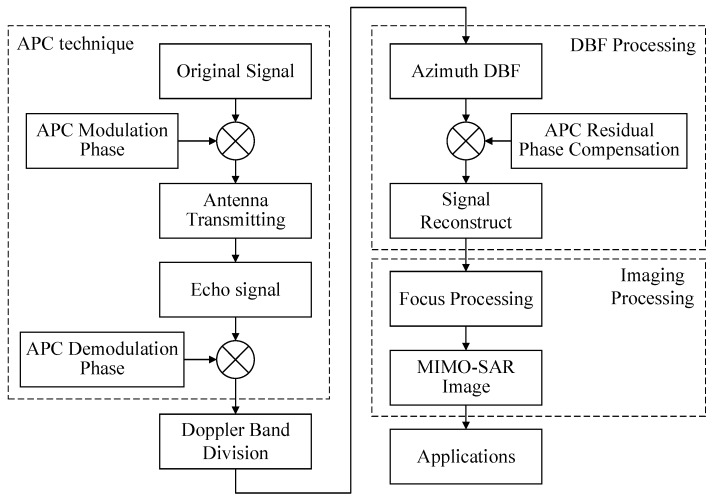
Flow chart of the proposed APC–MIMO–SAR solution.

For clarity, a useful flowchart of the proposed APC–MIMO–SAR solution is shown in Figure 8. The main steps can be summarized as the APC technique module, the DBF processing module and imaging processing module.

## 4. Discussion

In fact, the suggested approach, performing each Doppler frequency with a null-steering in the azimuth to reconstruct the unambiguous SAR signal, is closely connected to what has already been proposed in [35]. However, according to [35], any deviation from the multichannel displaced phase center aperture (DPCA) system or DPCA condition will deteriorate the performance of the null-steering and raise the noise level. In this section, the performance deterioration in the form of signal-to-noise ratio (SNR) needs to be discussed first in case of an unsatisfied DPCA condition.

Analogically, the DPCA condition in the suggested approach is

(19) $$X_{nm}-X_{n1}=\frac{2Nv}{f_{\mathrm{PRF}}}\left(\frac{m-1}{N}+k_{m}\right),\;k_{m}=[0,1,2,\ldots ]\;$$

where $X_{nm}$ denotes the azimuth coordinate of the Rxnm (m=2,3,…,M).

As a measure for the variation of SNR caused by the DBF network, the SNR scaling factor $\Phi_{DBF}$ [38] can be obtained by

(20) $$\Phi_{DBF}=\frac{SNR_{in}/SNR_{out}}{{\left(SNR_{in}/SNR_{out}\right)}_{\mathrm{DPCA}}}=N\sum_{k=1}^{N}E\left[{\left|a_{k}(f_{a})\right|}^{2}\right]\;$$

where SNR_in_ and SNR_out_ denote the SNRs before and after DBF, respectively. The operator E[·] represents the mean squared value operator, and $a_{k}(f_{a})$ is the k-th column in the matrix A in (16) in the Doppler frequency domain.

When the DPCA condition is satisfied, the optimum $\Phi_{DBF}$ is obtained. However, $\Phi_{DBF}$ will worsen with a rising mean squared value of $a_{k}(f_{a})$ due to the increased DPCA deviation.

To ensure a reliable echo separation by DBF, the formed Rx beam should be narrow enough, and thus the azimuth length of each receiving sub-aperture should exceed

(21) $$L_{a}\geq 2vK/\mathrm{PRF}\;$$

It should be emphasized that the sufficient receiving sub-apertures needed by echo separation require a longer azimuth antenna aperture, which may limit their application to the acquisition of additional phase centers and longer baselines in the azimuth.

## 5. Simulation Results

In this section, simulations on a point target and distributed targets are carried out to verify the validity of the proposed APC–MIMO–SAR solution based on the APC waveforms and echo separation method by azimuth DBF processing. The main parameters of a MIMO–SAR system are given in Table 1.

### 5.1. Simulation Results on a Point Target

In this part, a simulation is performed with a point target scene to show the effectiveness of the proposed Doppler spectra separation processing, using the azimuth digital beamforming (DBF) technique.

**Figure 9:**
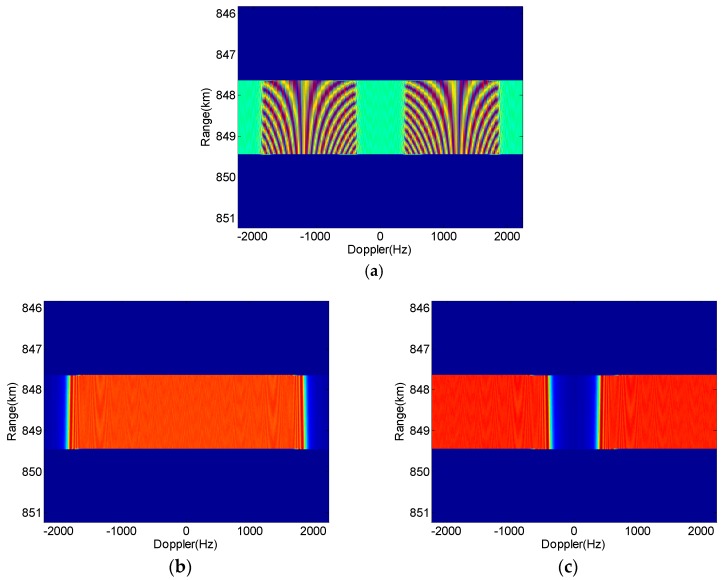
Doppler spectra of echoes of APC waveforms. (**a**) The overlapped Doppler; (**b**) the separated Doppler for Echo 1; (**c**) the separated Doppler for Echo 2.

From the experimental parameters in Table 1, one can notice that the PRF is set to be slightly larger than the Doppler bandwidth, which will cause the Doppler spectra aliasing occur after the sampling with PRF. Figure 9 shows the Doppler spectra of echoes before and after echo separation by azimuth DBF, in which the echoes are received by one of the four sub-apertures, for examplee Rx*11* For a better presentation in Figure 9, we assume the antenna pattern in the azimuth is in the rectangular shape. It is clearly observed in Figure 9a that the echoes of the two APC waveforms overlap each other because of the APC shift effect and the PRF sampling. After the Doppler sub-band-dependent azimuth DBF processing, the echoes are well separated, as shown in Figure 9b,c, where the Echo 2 shifts in the Doppler domain observably.

**Figure 10:**
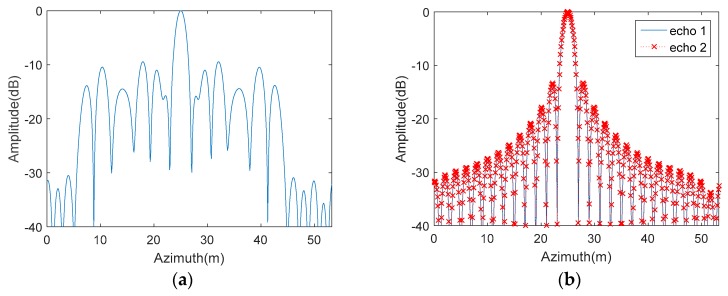
Azimuth imaging results (**a**) before and (**b**) after DBF.

Figure 10 presents the azimuth imaging results of the echoes received by Rx*11*. Figure 10a shows the focusing effect of the point target before the azimuth DBF processing, while Figure 10b shows the one after the processing. It is obvious that the focusing effect in Figure 10a is worse than that in Figure 10b. By comparing Figure 10a with Figure 10b, one can conclude that the focusing effect of the point target has been improved dramatically after the azimuth DBF processing.

### 5.2. Simulation Results on Distributed Targets

In this part, a simulation on distributed targets with the main parameters given in Table 1 is performed to show the advantage and the effectiveness of the proposed APC–MIMO–SAR.

**Figure 11:**
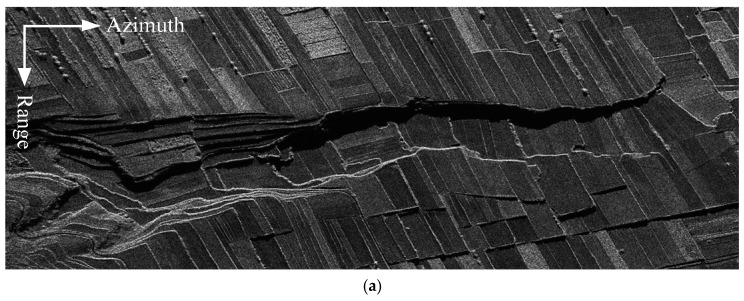

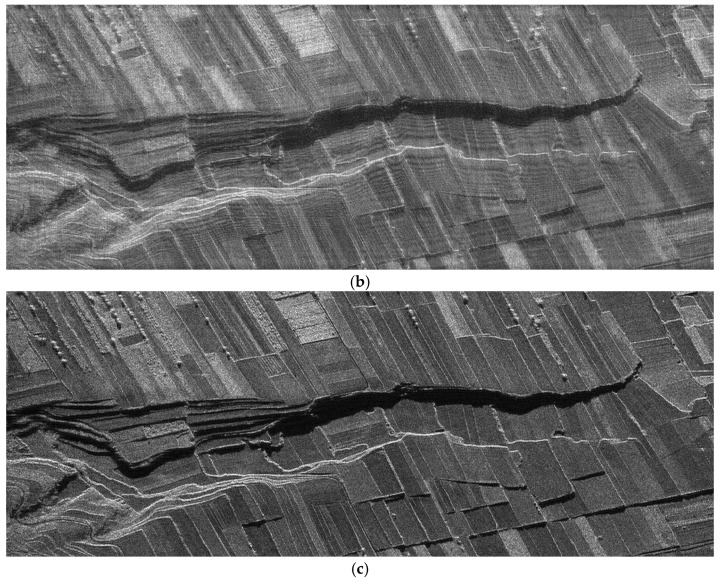
Simulation results of MIMO–SAR imaging varying with the transmitted waveforms. (**a**) Reference terrain scene. (**b**) Imaging result of up- and down-chirp waveforms. (**c**) Imaging result of the proposed APC waveforms.

Figure 11a shows a picture of the reference terrain scene that provides the input for the scene simulation. Figure 11b,c compare the focused images of the MIMO–SAR obtained via different transmit signals. Figure 11b shows the imaging result obtained via the conventional matched filter processing, when the MIMO–SAR sends an up-chirp signal and a down-chirp waveform simultaneously. From Figure 11b, one can notice that the leaked signal energy from the orthogonal waveform (down-chirp in this case) causes the degradation of the focusing performance obviously. Figure 11c shows the imaging result obtained via the DBF processing in the azimuth and the conventional matched filter processing, when the MIMO–SAR transmits the APC waveforms simultaneously. Figure 11c clearly demonstrates the good suppression of the mutual interference of the radar echoes by the proposed APC–MIMO–SAR solution. By comparing Figure 11b with Figure 11c, it is apparent that the quality of the whole image has been improved, and the focusing effect is better.

For a detailed imaging quality comparison, the image entropy and the contrast of the imaging results shown in Figure 11b,c are listed in Table 2. From Table 2, it is clear that the image entropy has decreased and the image contrast has increased in the proposed MIMO–SAR solution.

**Figure 12:**
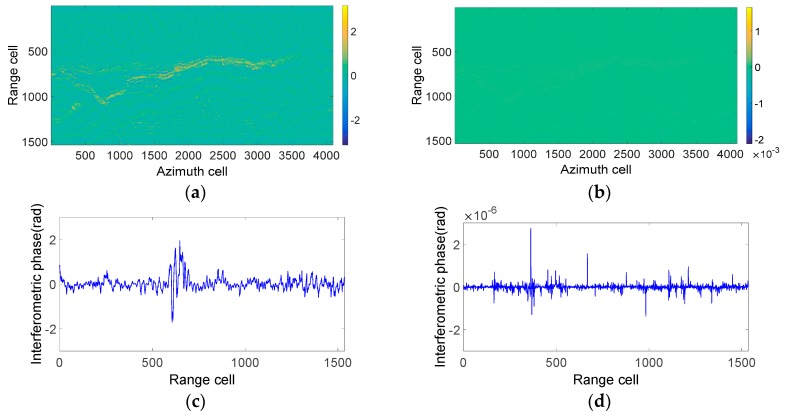
Simulation results of the interferometric phase between the reference terrain scene and (**a**) up- and down-chirp waveform MIMO–SAR and (**b**) the proposed APC–MIMO–SAR. (**c**) Middle azimuth cell of (**b**). (**d**) Middle azimuth cell of (**d**).

As the phase preservation of echo separation and reconstruction is very important in the multibaseline SAR system, the interferometric phase of the distributed targets has been analyzed in this section. Figure 12a shows the interference phase of the reference image (Figure 11a) and the complex image obtained by the up- and down-chirp waveforms (Figure 11b). The interference phase of the middle azimuth cell of Figure 12a is clearly shown in Figure 12c, which is up to 2 radians. From Figure 12a,c, it can be seen that the MIMO–SAR modulated by up- and down-chirp waveforms has poor phase-preserving performance. Figure 12b shows the interference phase of Figure 11a and the complex image obtained by the proposed APC–MIMO–SAR (Figure 11c). The interference phase of the middle azimuth cell of Figure 12b is close to 0 radians, as Figure 12d shows. From Figure 12b,d, one can see that APC–MIMO–SAR has better phase-preserving performance.

Based on the comparison of amplitude and phase shown in Figure 11 and Figure 12, the conclusion can be drawn that the proposed APC–MIMO–SAR imaging results are much closer to the ideal ones in both amplitude and phase, in contrast to MIMO–SAR transmitting up- and down-chirp waveforms. From this perspective, the proposed APC–MIMO–SAR has broader application prospects.

## 6. Conclusions

A novel MIMO–SAR solution, based on the principle of the APC technique combined with azimuth DBF, has been proposed in this paper. The proposed APC–MIMO–SAR provides a longer baseline in elevation, which will contribute more to the processing and application of multibaseline InSAR and three-dimensional SAR imaging. Applying the DBF processing in the azimuth as a spatial–temporal filtering to separate the aliasing echoes sustains a lesser PRF sampling, which will be beneficial to obtain a wider swath. Thus, the proposed APC–MIMO–SAR can be used to exploit the potentials of MIMO–SAR without performance degradation.

Furthermore, the good adaptability featured by APC waveforms allows them to combine with other waveforms easily, such as the multidimensional encoding waveforms or the orthogonal frequency division multiplexing (OFDM) waveforms. However, it is worth noting that the echo separation by azimuth DBF processing requires sufficient spatial degrees of freedom, which may lead to a longer azimuth aperture.

## Figures and Tables

**Table 1:** Main system parameters of simulations.

Parameters	Quantity	Parameters	Quantity
Wavelength	0.03 m	Signal bandwidth	100 MHz
Center frequency	10 GHz	Azimuth Doppler	3750 Hz
Sensor height	600 km	PRF	4500 Hz
Platform velocity	7500 m/s	Incident angle	45°
Transmitting aperture number	2	APC waveform number	2
Receiving sub-aperture number	4	Azimuth resolution	2 m

**Table 2:** The image entropy and the contrast of the imaging results.

Parameters	Entropy	Contrast
Figure 11b	7.1073	36.6054
Figure 11c	6.8526	115.3035
